# Study protocol for Project SHINE (Sleep Health INitiative for Equity): A community-based pilot RCT to promote sleep and physical activity among Black/African American adults

**DOI:** 10.1016/j.conctc.2025.101541

**Published:** 2025-08-29

**Authors:** Ivan HC. Wu, Lorna McNeill, Kristen Knutson, Yisheng Li, Diwakar Balachandran, Rhonda Jones-Webb, Pamela L. Lutsey, Darin Erikson, Shikha Bista, Rachel Price, Vanessa Anyanso, Taylor Smith, Rev Melvin Miller

**Affiliations:** a University of Minnesota – Twin Cities, School of Public Health, Division of Epidemiology and Community Health, USA; b University of Minnesota – Twin Cities, Masonic Cancer Center, USA; c University of Texas MD Anderson Cancer Center, Department of Health Disparities Research, USA; d Northwestern University, Feinberg School of Medicine, Center for Circadian and Sleep Medicine, USA; e University of Texas MD Anderson Cancer Center, Department of Biostatistics, USA; f University of Texas MD Anderson Cancer Center, Department of Pulmonary Medicine, USA; g University of Minnesota – Twin Cities, Department of Psychology, USA; h Progressive Baptist Church, Minneapolis, MN, USA

**Keywords:** Sleep, Health disparities, African americans, Physical activity, Community-based participatory research, Randomized controlled trial

## Abstract

**Background:**

Black/African American (AA) adults experience shorter sleep duration and poorer sleep quality compared to White counterparts, contributing to higher risks of chronic diseases. Project SHINE (Sleep Health INitiative for Equity) aims to address these sleep disparities by evaluating the feasibility, satisfaction, and plausibility (i.e., preliminary efficacy) of a culturally tailored sleep intervention designed to improve sleep duration and physical activity among AA adults with body mass index (BMI) ≥ 25 not meeting physical activity and sleep guideline recommendations.

**Methods:**

This pilot community-based randomized controlled trial (RCT) includes two phases. Phase 1 involves qualitative interviews with AA adults to explore sleep-related sociocontextual factors to refine the intervention. Phase 2 is an RCT (n = 80) assigning participants to a four-week sleep extension intervention or a contact control. The sleep extension intervention aims to improve sleep duration and physical activity. Sessions occur via Zoom, with in-person baseline and follow-up visits. Primary outcomes include feasibility and satisfaction of the intervention. Secondary outcomes include self-reported and objective sleep and physical activity measures, plus exploratory biomarkers for cancer and cardiovascular risk. Additional self-reports assess sleep-related psychosocial factors and health behaviors.

**Discussion:**

This study will assess the feasibility and implementation of a culturally tailored, virtual sleep intervention for AA adults. By integrating behavior change theories, cultural adaptation frameworks, and community-based participatory principles, *Project SHINE* aims to inform a larger-scale trial and support scalable behavioral interventions to improve sleep health and reduce disparities.

## 1 Introduction

African American (AA) adults experience disproportionately high rates of obesity, cardiovascular disease, diabetes, and cancer [1,2]. Short sleep duration has been shown to be a risk factor for obesity [[3], [4], [5]], cardiometabolic disease [6,7], diabetes [8,9], and cancer [[10], [11], [12], [13]], as well as systemic inflammation [14] and reduced physical activity [[15], [16], [17]]—both of which further contribute to chronic disease burden. AA adults also face significant sleep disparities, experiencing shorter sleep durations, poorer sleep quality, and longer sleep onset latency compared to White/European American counterparts [[18], [19], [20]]. Nationally, the prevalence of short sleep among Black adults increased from 35.3 % in 2004 to 41.7 % in 2018, widening the Black-White disparity from 7.51 to 10.68 percentage points [21]. In a community-based sample of AA adults recruited from churches in Houston, TX, nearly 61 % reported short sleep duration [3]. These disparities, shaped by socioeconomic, environmental, and psychological factors, likely exacerbate chronic disease prevalence in this population [22,23]. Despite strong evidence linking inadequate sleep to chronic illness, research on behavioral sleep interventions tailored for AA adults remains limited.

Given the association between inadequate sleep and excess weight-related chronic illnesses, targeted behavioral interventions to improve sleep quality and duration among AA adults could help reduce weight-related health disparities. Excess weight affects 40 % and 59 % of AA men and women, respectively [24], and is associated with 13 types of cancer. Despite public health campaigns promoting physical activity, most AAs (54.5 %) do not meet physical activity guidelines [25]. This inadequate activity increases the risk of excess weight, underscoring the need to target modifiable behaviors—such as sleep—to support healthier living.

Observational studies have shown that adequate sleep is associated with lower body mass index (BMI) and a reduced risk of excess weight [4,26]. Experimental studies further demonstrate that sleep restriction decreases physical activity and increases sedentary behavior [[15], [16], [17]], despite higher recorded activity counts due to increased wakefulness [27,28]. Clinical trials focused on sleep hygiene—such as maintaining consistent sleep schedules—have shown benefits including increased physical activity [29,30], reduced sedentary behavior [31], and improved dietary habits [32]. These trials also highlight the role of sleep in weight and fat mass loss [33,34], although findings are mixed, with some studies reporting limited effects on weight-related outcomes [35]. While these findings suggest that improving sleep could aid in weight management and reduce related chronic conditions within the AA community, most tailored sleep interventions focus primarily on sleep apnea [36,37] leaving a gap in research on behavioral sleep interventions [[38], [39], [40]]. It is important to note that while a recent meta-analysis found limited evidence for consistent bidirectional associations, analyses included only observational studies and exercise interventions without considering sleep interventions [41].

While the downstream effects of insufficient sleep highlight the need for intervention, understanding how upstream social and environmental factors shape sleep patterns among AA adults is essential for tailoring effective behavioral interventions. Broader socio-contextual factors can either promote or hinder sleep duration among AA adults [39,[42], [43], [44], [45]]. The majority of evidence highlights how environmental (e.g., neighborhood disadvantage [46,47] and stressful living conditions [48]) and individual-level factors (e.g., unfair treatment [49], chronic race-based discrimination [[50], [51], [52]], and microaggressions [53]) disrupt sleep initiation and maintenance among AA adults. Additionally, culturally-rooted concepts of resilience (e.g., Superwoman Schema and John Henryism [[54], [55], [56], [57], [58]]) play a complex role in shaping sleep health. While these beliefs foster strength and resilience, they can also lead to increased stress and emotional burden, which may exacerbate sleep issues. Despite extensive research on stress-related sleep disparities, protective factors remain underexplored. Studies have identified religiosity, spirituality [[59], [60], [61], [62], [63], [64], [65]], [[59], [60], [61], [62], [63], [64], [65]] and family [66] support as significant protective factors that foster a sense of community, reduce stress, and provide emotional and psychological support, which in turn may encourage better sleep quality and duration. This study aims to expand knowledge on both risk and protective factors influencing sleep health to refine a behavioral sleep intervention within a church-based setting.

The study is guided by the socioecological model of health [6,43,67,68], social cognitive theory (SCT) [69], and cognitive behavioral theory [70,71] to examine sociocontextual factors influencing sleep, physical activity, and weight status. These frameworks inform the cultural tailoring of a community-based behavioral sleep intervention. The *socioecological model* situates sleep within a broader context, recognizing it as an individual behavior shaped by environmental influences at multiple levels—individual, interpersonal, community, and societal—allowing for a holistic approach to sleep and health outcomes. *SCT* contributes a focus on the cognitive and social dimensions of behavior change, emphasizing how beliefs, attitudes, and perceptions about sleep are shaped by social environments. It also helps identify cognitive and social barriers and facilitators to adopting healthier sleep practices. *Cognitive behavioral theory* provides mechanisms for promoting behavior change and serves as the foundation for adapting selected components of Cognitive Behavioral Therapy for Insomnia (CBT-I). However, unlike full CBT-I, which includes sleep restriction and targets maladaptive beliefs related to insomnia, our intervention focuses on sleep extension for short-sleeping adults, emphasizing psychoeducation, stimulus control, and relaxation strategies to support gradual increases in sleep duration. This study is further grounded in Community-Based Participatory Research (CBPR) [72] principles, emphasizing collaborative partnerships, local relevance, and community engagement to ensure the intervention is culturally appropriate and beneficial to the community.

To address research gaps in community-engaged, culturally tailored efforts to reduce sleep disparities among AA adults, this pilot study will assess the feasibility, satisfaction, and plausibility of a pragmatic, virtually delivered behavioral sleep extension intervention. The study will proceed in two phases. Phase 1 (Aim 1) involves formative research and community engagement activities, including in-depth qualitative interviews with 10 AA adults to: (1) explore sleep-related social contextual factors, knowledge, behaviors, and beliefs, and (2) gather feedback on the intervention and study design. Phase 2 (Aim 2a and 2b) will consist of a randomized controlled trial. Aim 2a will evaluate the feasibility, satisfaction, and plausibility of the intervention in increasing sleep and physical activity among 80 AA adults with excess weight who do not meet physical activity or sleep duration guidelines, compared to a contact control group. Aim 2b will examine changes in cancer-relevant biomarkers (e.g., IL-6, TNF-α, CRP, fasting glucose, insulin, and adiponectin) in a subset of 20 participants.

## 2 Methods

**Overview*.*** Phase 1 focuses on conducting in-depth qualitative interviews to gain insights into sleep-related social contextual factors, knowledge, behaviors, and beliefs. This phase aims to gather feedback on the existing sleep intervention design and materials, ensuring they are culturally relevant and tailored to the needs of the community. Interviews will be conducted via Zoom to increase accessibility for participants. The collected data will help contextualize the lived experiences of the participants and inform the intervention to be implemented in Phase 2. Phase 2 involves a randomized controlled trial (RCT) to evaluate the feasibility, satisfaction, and preliminary efficacy of the sleep intervention compared to a contact control group. Eligible participants will be randomly assigned to one of two 4-week treatment conditions: the sleep extension intervention or the contact control. The sleep extension intervention aims to increase sleep duration by 1 h over four weeks, using components from CBT-I. Both intervention and control sessions will be conducted via Zoom. Based on recommendations for maintaining intervention fidelity [73], we will create an intervention manual, establish training and supervision protocols, and monitor both intervention delivery and participant engagement. We will regularly analyze fidelity data, provide corrective feedback, and document any deviations from the protocol to ensure the intervention is delivered as intended.

**Setting.** The study will be conducted in collaboration with Progressive Missionary Baptist Church in St. Paul, Minnesota, with all participant recruitment and in-person study activities occurring at this site, except for blood draws, which will take place at a university-affiliated facility. Phase 1 qualitative interviews and the 4-week intervention sessions for Phase 2 will be conducted virtually via Zoom to enhance accessibility. In Phase 2, baseline and follow-up assessments will be conducted in person at Progressive Missionary Baptist Church.

Progressive Missionary Baptist Church, led by co-author MM, has served the St. Paul community since 1992 and is widely recognized for its commitment to health equity and community engagement. With a predominantly Black/African American congregation of over 400 members, the church is affiliated with the Minnesota State Baptist Convention and the National Baptist Convention. Its ministry encompasses a range of programs, including youth development, discipleship, and pastoral care. The church holds weekly Sunday services in both virtual and in-person formats, with additional Bible studies and community activities occurring throughout the week. These services are recorded and broadcast online, further extending the church's outreach. Progressive Missionary Baptist Church has a history of engagement in health research and public health initiatives. At its initial outset, recruitment will be limited to congregation members.

**Recruitment and Eligibility Criteria.** The inclusion and exclusion criteria will be consistent across both Phases 1 and 2. The inclusion criteria are as follows: average habitual sleep duration of ≤6 h per night, not meet Physical Activity Guidelines for leisure physical activity (<150 min of moderate/vigorous activity or <75 min of vigorous activity), 21–75 years of age, Body Mass Index (BMI) > 25.0 kg/m^2^, and self-identify as Black or African American. Exclusion criteria include self-reported organ-related disorders such as COPD, cardiac arrhythmia, or gastro-esophageal disorder; being pregnant or less than four months postpartum; and having an infant under one year old living in the household.

The recruitment process will include flyers, attendance at community events, and announcements from church pulpits. Primary recruitment will initially occur at the community partner's site. Interested individuals will complete an electronic screener capturing demographic information, physical activity levels, sleep duration, and health status to determine eligibility. After completing the screener, participants will be contacted by study staff to notify them of their eligibility, receive an electronic consent form (eConsent) and an electronic questionnaire through REDCap. The research coordinator will then schedule a Zoom interview for Phase 1 participants or an in-person baseline visit for Phase 2 participants. To maintain engagement and retention, participants will receive reminders and follow-up communications from the research team. Participants who complete the screening and consent process will be enrolled in the study and scheduled for their respective activities, including interviews for Phase 1 and baseline assessments for Phase 2.

## 3 Phase 1: Pre-Interview Questionnaire, in-depth interview, and Analytic Plan (n = 10)

The primary goal of Phase 1 is to complete Aim 1, which is to better understand perceptions of sleep among AA adults and to gather feedback on an existing sleep intervention design. This phase involves conducting in-depth qualitative interviews to explore sleep-related social contextual factors, knowledge, behaviors, and beliefs. The aim is to contextualize the lived experiences of participants and identify themes related to the influence of multiple socioecological levels on perceptions of sleep. Eligible participants will be asked to complete an in-depth 60–90 min interview over Zoom. The semi-structured interviews explore a range of topics, including sleep problems, barriers and facilitators of sleep, sleep rituals, responses to inability to sleep, frequency of sleep-related discussions with health professionals, knowledge of health impacts of sleep, and how family, relationships, and the environment affect sleep. The purpose is to gather quantitative and qualitative data that reflects the diverse experiences and perspectives of the participants. Eligible participants will first complete a brief 10-min self-report questionnaire, followed by an in-depth interview. As compensation for their time and contribution, participants will receive a $50 gift card upon completion of the interview.

**Pre-Interview Questionnaire.** Participants will complete a self-report questionnaire prior to their in-depth interview. The demographic questionnaire will collect information on employment status, education level, household composition, alcohol use, sleep aid use, medical problems, and prior sleep disorder diagnoses. Sleep quality will be measured using the Pittsburgh Sleep Quality Index (PSQI) [74], with higher scores indicating poorer quality. Depressive symptoms will be assessed via the Patient Health Questionnaire (PHQ-9) [75], where higher scores indicate more severe depression. Neighborhood quality will be rated using the Neighborhood Problems Scale [76], and physical activity levels will be recorded with the International Physical Activity Questionnaire – Short Form (IPAQ) [77]. Church attendance will be measured by a single item on a six-point scale.

**In-depth Formative Interview.** The qualitative interview will explore participants' sleep habits, the impact of poor sleep on daily functioning and physical activity, and perceived sleep barriers and facilitators (see Table 1). It will also address the effects of local racial unrest on sleep, cultural factors influencing health and sleep disparities, culturally-based factors related to resilience (e.g., Superwoman schema [55], John Henryism [56]), and the relationship between spirituality and sleep. Given that recruitment for this study will take place within Progressive Missionary Baptist Church, questions will explore how the church and spirituality influence sleep and physical activity, as well as ways their church could better support healthy sleep and physical activity. As communities continue to recover from structural changes due to COVID-19 pandemic-related cultural shifts, we will also explore how the pandemic disrupted sleep patterns for many individuals due to heightened stress, changes in routines, and increased social isolation.

**Table 1:** Example formative interview questions**.**

Area	Questions
Social	How does work, environment, pets, children, and family impact your sleep? In what ways does your faith and the church (e.g., attendance) impact your sleep?
Context	
Knowledge	How much sleep do you think you need? What have you heard about sleep hygiene recommendations?
Behavior	What gets in the way of getting enough sleep? What do you do before going to bed?
Beliefs	How does inadequate sleep affect your health? How does your sleep affect your PA or exercise? Do women and men have similar experiences with sleep?
Sleep	How can content be more relevant to the AA community? How can the intervention be improve to make it more acceptable?
Intervention	

**Analytic Plan.** Analytic Plan: In Phase 1, qualitative data from the in-depth interviews will be analyzed using NVivo software to organize and code the data, identifying common themes, patterns, and variations in participants' responses. The coding team, consisting of one PhD research associate, two PhD-level graduate students, and two post-baccalaureate students—all of whom with lived experience as a racial/ethnic minority in the US—will use a combined inductive and deductive thematic analysis approach [78]. Deductive codes will be generated based on pre-determined theories and research questions, while inductive codes will emerge organically using a grounded theory approach.

Before coding begins, the team will jointly code a transcript to ensure consistency. For each subsequent transcript, a primary, secondary, and tertiary coder will be assigned. The primary coder will conduct multiple readings of the transcript: first to familiarize themselves with the content, then to take notes, and finally to develop preliminary codes. The secondary coder will read the transcript, generate additional codes and notes, and identify any areas missed by the primary coder. The tertiary coder will review all quotes assigned to the codes to ensure they align, noting any discrepancies. The team will then convene to discuss the codes and collaboratively develop themes. Any discrepancies will be addressed and resolved during these meetings.

## 4 Phase 2: intervention, adaptation, implementation, and Analytic Plan (n = 80)

**Fig. 1:**
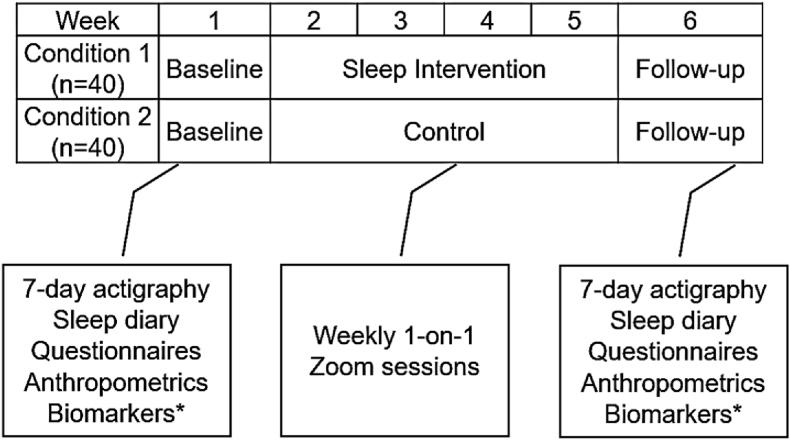
Aim 2 sleep intervention randomized control trial design. *∗*Measurement of metabolic (insulin, adiponectin, leptin) and pro-inflammatory (IL-6, CRP, TNF-α).

During Phase 2, we will complete Aim 2a and 2b of the study, which focuses on exploring the feasibility, satisfaction, and plausibility (i.e., preliminary efficacy) of a sleep intervention designed to increase sleep duration and physical activity (PA). This phase employs a 6-week randomized controlled trial (RCT) to evaluate the intervention compared to a contact control group (see Fig. 1). Phase 2 involves a 7-day baseline assessment, followed by a 4-week intervention period, and concluding with a 7-day follow-up assessment. Eligible participants will be randomly assigned to one of two conditions: a sleep extension intervention or contact control. The primary goal of the sleep intervention is to increase sleep duration by 1 h over the course of four weeks and improve PA.

**Interventions.** The sleep extension intervention is designed for AA adults who habitually obtain insufficient sleep, regardless of sleep disorder status, and aims to increase nightly sleep duration and daily physical activity. While sleep duration is a primary target for preliminary efficacy, we include physical activity based on prior research indicating the reciprocal relationship between sleep and physical activity (see *Introduction*). The intervention adapts selected components of Cognitive Behavioral Therapy for Insomnia (CBT-I) [79], including psychoeducation and stimulus control, while intentionally excluding sleep restriction. Participants receive four weekly one-on-one sessions via Zoom, delivered by trained study staff following a standardized protocol. Interventionists include master's-level counseling psychology graduate students trained in behavioral sleep principles and community engagement. Sessions follow a structured framework. Session 1 (60 min) introduces foundational sleep health concepts, including psychoeducation on the physiological and cognitive impacts of insufficient sleep, relaxation training, and individualized goal setting for bedtime extension. Sessions 2–4 (30 min each) involve review of weekly sleep diaries, monitoring of adherence to incremental bedtime targets (e.g., 15-min weekly increases), identification of barriers, and reinforcement of sleep-promoting behaviors such as consistent wake times and reduced evening screen use. Culturally relevant strategies—such as faith-based wind-down routines and household sleep negotiations—are integrated based on participant context. Tailored home practice recommendations, including relaxation exercises, are also provided weekly. Participants are encouraged to maintain their extended sleep schedule during the follow-up week. The intervention does not include any content related to physical activity promotion or goal-setting. However, physical activity is assessed as a secondary, non-targeted behavioral outcome to examine whether increasing sleep duration may be associated with incidental changes in activity levels.

The structure of the intervention limited to 4 sessions was based on a meta-analysis of 87 CBT-I randomized controlled trials (RCTs), which concluded that a minimum of 4 sessions was necessary for meaningful improvements in sleep outcomes compared to self-help interventions or fewer sessions [80]. Further, evidence suggests that short, pragmatic community-based workshops can significantly improve sleep efficiency and reduce insomnia symptom severity [81], reinforcing the potential effectiveness of a brief intervention approach. However, given that this intervention targets insufficient sleep rather than clinical insomnia, its structure reflects a pragmatic adaptation aimed at improving sleep duration in a population that may not necessarily meet diagnostic criteria for insomnia.

The contact control condition is based on the National Center for Healthy Housing's Healthy Homes program, designed to control for time, engagement, and participant interaction with study staff. Participants in this group will receive four weekly Zoom sessions (15–30 min each) covering topics such as indoor air quality, emergency preparedness, pest control, injury and fall prevention, and poison control. The sessions parallel the sleep intervention in frequency and duration but focus on home health education rather than sleep behavior change. Staff will also check in on participants' progress in applying the educational content and address any challenges.

**Training, Supervision, and Fidelity Monitoring.** All counselors delivering the interventions are graduate students in counseling or clinical psychology with prior clinical experience and foundational training in psychotherapy theories. The study PI (IW), a trained clinical psychologist with formal training in CBT-I, will oversee counselor training and supervision. Interventionist training is delivered across three structured sessions and covers: (1) the theoretical framework and content of the sleep extension intervention, (2) delivery of the contact control (Healthy Homes) condition, and (3) use of REDCap for intervention delivery, data entry and fidelity tracking. Training includes didactic instruction, in-session modeling, and supervised practice of all intervention components. Counselors also conduct mock sessions with test cases and in-person rehearsals with the PI prior to implementation. Counselors attend weekly team meetings for supervision, where they will present and receive individualized case feedback from the PI. Supervision focuses on reviewing participant progress, identifying barriers to implementation, and ensuring intervention adherence. All intervention sessions will be audio-recorded (with participant consent), and a subsample reviewed periodically by the PI. Fidelity is monitored using a REDCap-based session checklist that tracks delivery of core content and session structure. Each session will be scored based on the percentage of protocol elements delivered, with a threshold of ≥80 % considered acceptable fidelity. If a counselor delivers fewer than 80 % of required elements in more than one reviewed session, they will receive targeted retraining. Retraining will include direct supervision with the PI, review of fidelity checklists, and additional roleplay-based practice on missed intervention components.

**Adaptation*.*** The adaptation of the sleep intervention will follow a modified version of the Integrated Model of Cultural Adaptation of Evidence-Based Interventions [82]. This model provides a structured framework for systematically incorporating cultural relevance while maintaining intervention fidelity. Key stages include gathering evidence to assess adaptation needs, incorporating qualitative and quantitative data, and integrating community feedback to refine study materials before full-scale implementation. Community input will be obtained through multiple sources, including qualitative interviews from Aim 1, discussions with community leaders, and a Community Advisory Board (CAB) consisting of gender- and age-diverse participants, will identify culturally relevant adaptations and ensure the intervention aligns with community needs and values. Regular meetings with the CAB will be co-facilitated by the principal investigator and an experienced community member to ensure discussions reflect community priorities and perspectives.

The adaptation process will address both surface structure and deep structure elements of the intervention [83]. Surface structure adaptations focus on presentation and accessibility, such as collaborating with community members to design culturally appropriate study flyers, recruitment materials, and outreach strategies for community events. Deep structure adaptations ensure that the intervention reflects community values, historical experiences, and cultural beliefs. These adaptations may include: (1) conducting data collection at the church in addition to the university to acknowledge its cultural and historical significance and address hesitations stemming from historical mistrust of university-based research; (2) integrating culturally relevant resilience frameworks, including the Superwoman Schema [54,55] and John Henryism [56,57] to acknowledge the community's unique stress experiences and coping strategies; (3) incorporating faith-based practices [84] and social support systems to enhance participant engagement and reinforce existing cultural resources; and (4) including congregation members with clinical experience in the intervention delivery and coding team, fostering trust and community ownership over the research process. By integrating culturally specific conceptualizations of stress and resilience, these adaptations enhance the intervention's relevance and acceptability while fostering greater engagement and trust within the community [[85], [86], [87], [88], [89], [90], [91]].

A Community Advisory Board (CAB) will be established composed of congregation members from the community partner's church. The CAB will meet more frequently during the initial implementation phase and transition to quarterly meetings during the active intervention phase. The lead pastor will participate in quarterly study team meetings to ensure ongoing alignment with church priorities. Two congregation members will serve as core members of the research team and will attend all team meetings. They will provide real-time feedback during implementation planning and decision-making and will also assist in organizing and facilitating CAB activities. CAB members will review intervention materials, suggest culturally relevant adaptations, inform recruitment and retention strategies, and help interpret preliminary findings for community-facing dissemination. This structure will ensure sustained, bidirectional engagement and accountability between the academic and church partners across all phases of the study.

**Implementation (randomization and assessment periods).** At the end of the baseline visit, participants will be randomized into one of the two conditions using minimization randomization,{Scott, 2002 #129} taking into account participants’ sex assigned at birth. The baseline assessment includes a 7-day period where participants complete self-administered questionnaires, wear an accelerometer to monitor activity and sleep, and undergo a blood draw if they volunteer for this component (n = 20). After the 4-week intervention period, a follow-up assessment identical to the baseline assessment will be conducted. This includes completing questionnaires, wearing the accelerometer for another 7-day period, and undergoing a second blood draw for those who volunteered. Retention strategies include regular follow-up communications and reminders from the research team. Participants will receive financial compensation for their participation: a gift card for each in-person assessment visit ($50 for the baseline visit, $50 for the follow-up visit, and $50 for returning the actigraph watch). Those volunteering for blood draws will receive additional compensation ($40 for the first visit, $60 for the second visit).

**Measures.** Participants will complete both self-report questionnaires (e.g., measures of social contextual factors, psychosocial factors, and health behaviors), as well as physical measurements (e.g., anthropometric and blood draws).

*Primary Outcomes.* Primary outcomes include feasibility and satisfaction of the culturally adapted sleep extension intervention. Feasibility will be determined by achieving an average intervention adherence rate of at least 75 % among participants in the sleep intervention group. That is, feasibility is achieved if participants attend an average of at least 3 out of 4 sessions. Adherence is operationalized as the proportion of sessions attended per participant, averaged across all participants in the intervention group. The adherence rate is the proportion of sessions attended per participant, averaged across the intervention group. Satisfaction will be assessed through participant interviews and the Client Satisfaction Questionnaire [92] that measures how well the intervention met the participants' needs, perceived improvement in skills, and likelihood of recommending intervention to others). Higher scores indicate greater satisfaction with the intervention, and satisfaction will be met if average scores across participants is ≥ 20.

*Secondary outcomes.* Secondary outcomes include objective and subjective measures of physical activity and sleep, as well as cancer risk-related biomarkers pre- and post-intervention. Although the intervention does not directly target physical activity, we assess PA to explore whether increasing sleep duration may yield subsequent changes in movement patterns. Self-reported physical activity levels will be assessed with the 7-item International Physical Activity Questionnaire – Short (IPAQ-SH) [93], and sedentary behavior will be assessed using a single-item measure of sitting used in National Health and Nutrition Examination Survey (NHANES). Self-reported sleep duration and quality will be measured using the 19-item Pittsburgh Sleep Quality Index (PSQI) [74], and the 9-item Consensus Sleep Diary [94]. Objectively measured PA and sleep duration and quality will be measured using wrist-worn actigraphy on the non-dominant hand for 1-week pre/post-intervention. *Exploratory biomarker* outcomes (n = 20) include pro-inflammatory markers (IL-6, TNF-α, CRP) and metabolic indicators (insulin, glucose, adiponectin), and blood pressure.

*Sleep-related psychosocial and health behavior questionnaire.* A number of sleep-related social psychosocial and health behavior questions will be completed pre- and post-intervention to contextualize participants’ lived experiences. Social contextual factors will assess the following: family functioning (Family APGAR Scale) [95], perceived social support (Interpersonal Support Evaluation List; ISEL) [96], church attendance and participation church ministries, work stress (Job Content Questionnaire; Decision Latitude and Psychological Demands subscales) [97], and perceived neighborhood safety and quality [76]. Psychosocial factors will include measures of perceived stress (Perceived Stress Scale; PSS) [96], spirituality [98]; discrimination (Day-to-Day Unfair Treatment Scale) [51], financial stress, and perceived social status (Social Status Ladder [99]). Lastly, health behaviors will include measures of perceived sleep control (Brief Index of Sleep Control [100]), sleep apnea risk (STOP-BANG [101]), insomnia severity (Insomnia Severity Index; ISI [102]), Restless Leg Syndrome [103], diet (Food Frequency Questionnaire; FFQ [104]), as well as tobacco and alcohol use [105].

*Anthropometric measurements* will be collected pre- and post-intervention by trained staff, including height, weight, neck, hip, waistline, and body fat percentage.

**Sample size justification for Phase 2**. The sample size of 80 participants (40 per group) was selected to allow for reasonable estimation of effect sizes and 95 % confidence intervals (CIs) to inform future trials. While this study is not designed for formal hypothesis testing, a power analysis was conducted to assess the ability to detect a potential Time × Condition interaction on sleep and physical activity (PA). G∗Power version 3.September 1, 7 [106]. was used with the following parameters: α = .05, two groups (intervention and control), two measurements (pre and post), and an intra-class correlation (ICC) of .50 to provide a more conservative estimate. Effect size estimates for the f-statistic were varied as follows: small (f = .10), moderate (f = .25), and large (f = .40). Based on these parameters, a sample size of 80 participants (40 per condition) provides 80 % power to detect a moderate-to-large effect size (f ≈ .35), accounting for a 12.5 % attrition rate.

**Analytic plan.** Quantitative data from Phase 2 will be analyzed to assess feasibility, acceptability, and preliminary efficacy. Descriptive statistics, including means, medians, standard deviations, and 95 % confidence intervals (CIs), will be reported for sleep and PA outcomes. Pre-post changes will be examined for ceiling or floor effects and range restrictions. Graphs will visualize outcome distributions over time by group, highlighting differences in demographics and retention status.

While the power analysis was originally conducted for a repeated measures ANOVA, the primary analytic approach will use linear mixed models (LMMs) to account for repeated measurements and missing data. Given the exploratory nature of this pilot study, p-values will not be reported. Instead, standardized effect sizes (e.g., Cohen's d for between-group differences, standardized β coefficients from LMMs) will be reported with their standard deviations and 95 % CIs to evaluate trends and inform future trials.

LMMs will be used to examine within-group and between-group changes in primary outcomes (sleep quality, sleep duration, PA, and sedentary behavior) over the 4-week study period. While group differences over time will be explored, this study is not powered to detect interaction effects with high precision. Therefore, results will focus on descriptive effect size estimates rather than hypothesis-driven interaction testing. Analyses will follow the intention-to-treat (ITT) principle, ensuring that all randomized participants are included in analyses regardless of adherence. Missing covariate data will be handled via multiple imputation, while missing outcome data will be addressed using maximum likelihood estimation (MLE) within the LMMs. Baseline differences in participant characteristics and outcomes will be examined, with significant differences included as covariates to control for potential confounding. Loss to follow-up rates and differential attrition effects will also be evaluated.

For exploratory Aim 2b, changes in inflammatory and metabolic biomarkers (or their log transformations, if needed) will be described using means, standard deviations, and 95 % CIs to identify trends in proinflammatory and metabolic markers.

## 5 Discussion

Project SHINE is a community-based pilot study designed to address significant sleep disparities among Black/African American adults. This study evaluates the feasibility, satisfaction, and plausibility (i.e., preliminary efficacy) of a culturally tailored, virtually delivered sleep intervention aimed at improving sleep duration and physical activity. Although physical activity behaviors were not targeted during the intervention sessions, we measure it as a secondary outcome to assess whether improvements in sleep may indirectly support increased movement. The sleep extension intervention adaptation and pilot feasibility study is structured into two phases. Phase 1 involves qualitative interviews to explore sleep-related social contextual factors and refine the intervention. Phase 2 is a randomized controlled trial comparing the sleep extension intervention to a contact control group. This trial will provide insights into the contextual factors affecting the adoption of behavioral sleep interventions within a church setting and identify barriers and facilitators to implementation. Additionally, it will generate data to estimate effect sizes, informing the design of a future efficacy trial. The findings will contribute to the development of pragmatic community-based behavioral interventions to improve sleep among AA adults.

## Ethics approval and clinical trial registration

The trial was granted full ethics approval by the University of Minnesota Institution Review Board (Reference number STUDY00017901). The trial is registered with ClinicalTrials.gov ID: NCT06226077.

## CRediT authorship contribution statement

**Ivan HC. Wu:** Writing – review & editing, Writing – original draft, Supervision, Project administration, Methodology, Investigation, Funding acquisition, Formal analysis, Conceptualization. **Lorna McNeill:** Writing – review & editing, Conceptualization. **Kristen Knutson:** Writing – review & editing, Supervision, Methodology, Conceptualization. **Yisheng Li:** Writing – review & editing, Formal analysis, Conceptualization. **Diwakar Balachandran:** Methodology, Conceptualization. **Rhonda Jones-Webb:** Writing – review & editing, Supervision, Methodology. **Pamela L. Lutsey:** Writing – review & editing, Supervision, Project administration, Methodology. **Darin Erikson:** Writing – review & editing, Supervision, Project administration, Formal analysis. **Shikha Bista:** Writing – review & editing, Writing – original draft, Formal analysis. **Rachel Price:** Writing – review & editing, Writing – original draft, Project administration. **Vanessa Anyanso:** Writing – review & editing, Writing – original draft, Investigation, Formal analysis. **Taylor Smith:** Writing – review & editing, Writing – original draft, Project administration, Investigation. **Rev Melvin Miller:** Resources, Investigation.

## Funding

Funding support for this work was provided by the National Institutes of Health (R00MD015296 and P30CA077598).

## Declaration of competing interest

The authors declare there are no known competing financial interests or personal relationships that could have appeared to influence the work reported in this paper.

## Data Availability

Data will be made available on request.
